# Respiratory events in ward are associated with later intensive care unit (ICU) admission and hospital mortality in onco-hematology patients not admitted to ICU after a first request

**DOI:** 10.1371/journal.pone.0181808

**Published:** 2017-07-27

**Authors:** Laure Doukhan, Magali Bisbal, Laurent Chow-Chine, Antoine Sannini, Jean Paul Brun, Sylvie Cambon, Lam Nguyen Duong, Marion Faucher, Djamel Mokart

**Affiliations:** Intensive Care Unit, Institut Paoli Calmettes, Marseille, France; National Yang-Ming University, TAIWAN

## Abstract

**Introduction:**

Prognostic impact of delayed intensive care unit(ICU) admission in critically ill cancer patients remains debatable. We determined predictive factors for later ICU admission and mortality in cancer patients initially not admitted after their first ICU request.

**Methods:**

All cancer patients referred for an emergency ICU admission between 1 January 2012 and 31 August 2013 were included.

**Results:**

Totally, 246(54.8%) patients were immediately admitted. Among 203(45.2%) patients denied at the first request, 54(26.6%) were admitted later. A former ICU stay [OR: 2.75(1.12–6.75)], a request based on a clinical respiratory event[OR: 2.6(1.35–5.02)] and neutropenia[OR: 2.25(1.06–4.8)] were independently associated with later ICU admission. Survival of patients admitted immediately and later did not differ at ICU(78.5% and 70.4%, respectively; *p* = 0.2) or hospital(74% and 66%, respectively; *p* = 0.24) discharge. Hospital mortality of patients initially not admitted was 29.7% and independently associated with malignancy progression[OR: 3.15(1.6–6.19)], allogeneic hematopoietic stem cell transplantation[OR: 2.5(1.06–5.89)], a request based on a clinical respiratory event[OR: 2.36(1.22–4.56)] and severe sepsis[OR: 0.27(0.08–0.99)].

**Conclusion:**

Compared with immediate ICU admission, later ICU admission was not associated with hospital mortality. Clinical respiratory events were independently associated with both later ICU admission and hospital mortality.

## Introduction

Triage decisions concerning intensive care unit (ICU) admission or continued management of wards is subject to intensivists’ assessments. Rapidity of care in critical situations may have a direct impact on survival [1]. To confront in-hospital emergency situations, rapid response teams (RRTs) have been implemented in many hospitals [2]. The situations in which patients remain in the ward following RRT assessment frequently occur. A later evaluation may be needed, and ICU admission could then be decided after one or several subsequent referrals [3]. This raises the question of the impact of such a ‘later admission’ compared to that of an ‘immediate admission’.

Among patients not admitted at the first RRT evaluation, we aimed to identify factors associated with an increased risk of later admission compared with those in patients never admitted to ICU and to assess the prognostic impact of such a later admission compared with an immediate admission. Secondary objectives included the identification of factors associated with hospital mortality present at the time of the first RRT call as it led to a non-admission and gain knowledge on the outcome of patients never admitted.

## Patients and methods

### Study framework

The study was conducted at the ‘Institut Paoli-Calmettes’, a comprehensive cancer centre in Marseille (France), where approximately 10,000 patients are hospitalised every year. The institute has four haematology units, including a bone marrow transplantation unit, three medical oncology units, two surgical oncology units and one ICU. ICU is composed of eight intensive-care beds, seven intermediate care beds and one additional bed for life-threatening situations.

### Patients

All emergency ICU requests concerning admission or advice from 1 January 2012 to 31 August 2013 were prospectively included. Planned admissions in the context of postoperative surveillance were not considered. In case of multiple requests and/or multiple ICU stays for the same patient, only the first event was analysed. Initially, patients not admitted were divided into two groups: the ‘later admission’ group defined as a delayed ICU admission after subsequent request(s) during the same hospitalisation and the ‘no admission’ group defined as no ICU admission between the first RRT call and the end of the hospitalisation. These two groups extracted from a pool of patients initially not admitted represented our study population. Hospitalised patients who were immediately admitted after the first request and patients coming from outside the institute and directly admitted to ICU formed the control group, named ‘immediate admission’. The institutional review board of the ‘Institut Paoli–Calmettes’ approved the study and waived the need for informed consent due to the observational nature of the study.

### Definitions

A former ICU admission was defined as a recent (< 3 months) ICU admission not during the ingoing hospitalization. A later ICU admission was defined as a subsequent ICU admission during the same hospitalization, following at least a first request for which no ICU admission was decided. Respiratory, haemodynamic and neurological clinical events were composed of one or several items (Table 1). Hypoxemia was defined as an oxygen saturation of <90% on room air. Dyspnoea was defined as a difficulty in breathing at rest and/or a respiratory rate of >30 breaths per min and/or clinical signs of respiratory distress. Acute respiratory failure (ARF) was defined as oxygen saturation of <90% or PaO2 of <60 mmHg on room air combined with severe dyspnoea at rest combined with inability to speak in sentences or a respiratory rate of >30 breaths per min or clinical signs of respiratory distress [4]. Hypotension (systolic blood pressure < 90 mmHg or a decrease amounting to >40 mmHg), severe sepsis (acute organ dysfunction secondary to documented or suspected infection) and shock (severe sepsis plus hypotension not reversed by fluid resuscitation) were defined according to the criteria of the Surviving Sepsis Campaign [5]. Acute kidney injury was defined according to the AKIN classification scheme[6] as either i) serum creatinine levels of ≥26.4 μmoL/L (0.3 mg/dL) occurring within 48 h, ii) an elevation in serum creatinine levels to ≥150% from baseline or iii) a urine output of <0.5 mL/Kg/h for 6 h or more. A ‘warning’ was requested by the ward physician when he judged a patient to be at risk of deteriorating in the hours or days ahead. ‘Simple medical advice’ concerned non-emergency situations in which the ward physician asked the senior intensivist for advice. Neutropenia was defined as an absolute neutrophil count of <0.5 x 10^9^/L. Control of the underlying malignant disease was categorised into five levels: controlled disease (response to therapy), complete remission (normalisation of bone marrow and peripheral blood counts for haematologic malignancies; absence of any clinical, biological or radiological sign of solid malignancy after treatment), initial phase of treatment (cancer diagnosed within the past four weeks), ongoing treatment (with no definition of a therapeutic response) and disease in progression (pejorative evolution despite curative treatment or palliative management).

**Table 1:** Reasons for ICU requests among initially not admitted patients.

	N	(%)
**Clinical respiratory event**	**92**	**(45.3)**
Hypoxemia	66	
Dyspnea	40	
Acute respiratory failure	19	
Bad tolerated pleural effusion	5	
Acute asthma	1	
**Clinical hemodynamic event**	**83**	**(40.9)**
Hypotension	35	
Severe sepsis	26	
Cardiac arrythmia or conducion disorders	23	
Urine output < 0.5 ml.kg-1.h	11	
Bleeding	6	
Cardiac arrest	5	
Shock statement	4	
**Clinical neurological event**	**31**	**(15.3)**
Coma	4	
Confusion	15	
Convulsions	7	
Other neurological disorder	5	
**Alteration of renal function or metabolic abnormality**	**23**	**(11.3)**
Acute kidney injury (AKIN classification)	17	
Ionic trouble	7	
**Warning**	**22**	**(10.8)**
**Unspecified reason**	**11**	**(5.4)**
**Simple medical advice**	**8**	**(3.9)**

### Role of RRT and ICU admission

RRT involves senior intensivists and is reachable 24 h a day and 7 days a week through a dedicated number. The team provides immediate help concerning in-hospital life-threatening situations or simple medical advice. During off hours, defined as the period from 6 pm to 8 am, weekends and national holidays, a single senior intensivist is on duty to take care of ICU patients and to assure the RRT service, whereas during office hours, defined as the period from 8 am to 6 pm during weekdays, three full-time physicians are present. Decisions to admit patients to ICU were made by referring oncologists or haematologists and a senior intensivist. ICU admission is usually considered when a patient has at least one organ dysfunction. Some patients are admitted for inaugural malignancies with a view to initiating treatment to reduce the risk organ failure: malignancies involving bulky tumours (essentially lymphoma) or patients aged ≤70 years presenting with acute leukaemia with both leucocytosis (>50 x 10^9^/L) and thrombocytopenia (<50 x 10^9^/L) at the time of diagnosis. After clinical examination, patients were considered to stay in wards if they presented with spontaneous respiration with oxygen need ≤ 4l O2/min with no need for non-invasive mechanical ventilation therapy; stable hemodynamics with no use of vasoactive drugs; no relevant neurologic impairment; no increased serum lactate; no significant (< 2mg/dl)blood loss during the last 24h. During the ICU stay, life-supporting interventions, anti-infectious agents, prophylactic treatments, urate oxidase use and diagnostic procedures were administered at the discretion of the attending intensivists, who followed best clinical practice and guidelines [5]. Chemotherapy, corticosteroids, haematopoietic growth factors, immunosuppressive drugs and other cancer-related treatments were prescribed by the haematologist/oncologist in charge of each patient in accordance with institutional guidelines [5]. Decisions on ICU discharge were left at the discretion of the intensivists, and patients were discharged from ICU without any non-haematological organ failure. Patients remaining in the ward following RRT assessment were managed daily by both the physician in charge of the patient and the intensivist, who could offer medical advice and eventually propose to introduce new or modify ongoing therapeutics.

### Data collection

For each non-admission throughout the study period, the on-call RRT intensivist prospectively filled out a register specifying information concerning the time and provenance of the request, the reason for it and the reason for the non-admission. The reasons for requesting could have variable degrees of severity and are listed in Table 1. There could be several reasons for a single request. A simple medical advice was defined as medical advice not requiring an ICU hospitalisation. An unspecified reason was defined as a request for which the reason was not collected in the register. Reasons for non-admission could be a patient considered ‘too well to benefit’ from an ICU admission, a patient considered ‘too sick to benefit’, or the lack of bed availability. Data in the tables and figures were prospectively collected. The Simplified Acute Physiology Score II [7] and Charlson comorbidity index [8] were computed at ICU admission. These scores provide an estimate of the risk of death based on patient characteristics at admission. The last follow-up date was set on 15 August 2014.

### Follow-up

The patients were followed up for at least one year using the electronic system available at the hospital. The electronic system is used for administrative and medical purposes in all services and procedures, and visits, laboratory examinations, vital signs and other data gathered during hospitalization or outpatient visits are consistently recorded along with the date and a unique identifier. The Institut Paoli-Calmettes has a policy of following its patients, and as a general rule, at least one scheduled visit every three months is required for cancer patients to be discharged. During the study period, three patients were not treated in our ICU but were transferred to other ICUs. For these patients, some ICU parameters were not included in the analysis because of data unavailability. Among these patients, one patient was lost to follow-up on ICU discharge.

### Statistical analysis

Categorical data were reported as percentages and analysed using chi-squared test or Fisher’s exact test, according to the sample size. Continuous variables were reported as medians and quartiles (interquartile ranges) and analysed using the Mann–Whitney U test or Wilcoxon signed rank test for related samples. To identify predictive factors of a later admission among patients initially not admitted, the groups ‘later admission’ and ‘no admission’ were compared. To identify factors associated with hospital mortality present at the time of the first request, two others groups were developed from the patients initially not admitted according to their vital status at hospital discharge. Univariate analysis and multivariate logistic regression analysis were performed for these two objectives. A p-value of <0.05 was considered statistically significant. Statistically significant variables after univariate analysis and clinically relevant variables were entered into the multivariate analysis.

Kaplan–Meier survival curves were plotted, and the log-rank test was used to compare the ‘later admission’ group with the ‘immediate admission’ group and the patients initially not admitted presenting with respiratory events with those not presenting with respiratory events.

Statistical tests were performed using the SPSS statistics software package (SPSS, Version 16.0 Chicago, IL, USA).

## Results

### Characteristics of patients at the first request

**Fig 1:**
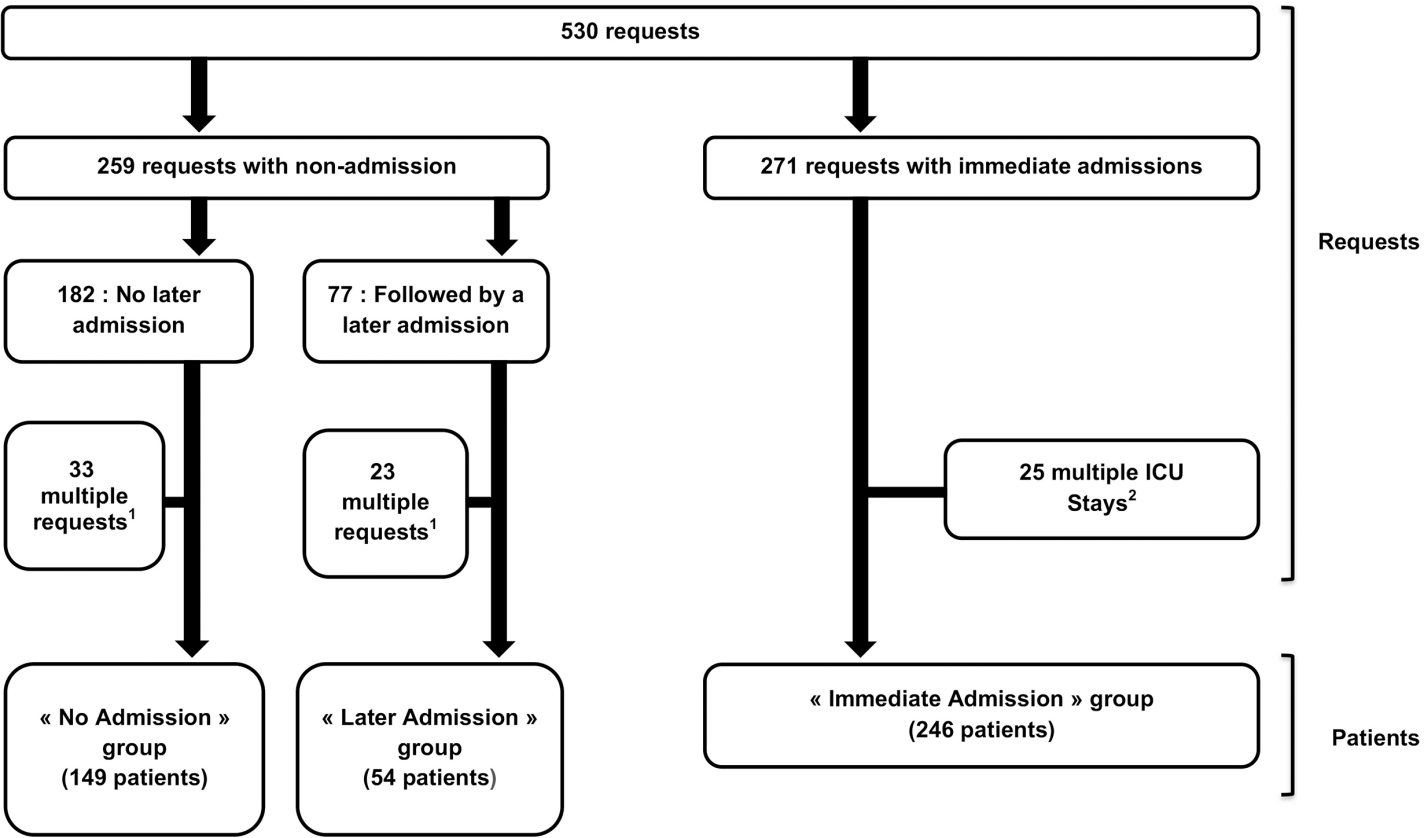
Flow chart. ^1^ Multiple requests for a same patient: only the first was analysed. ^2^ Multiple ICU stays for a same patient: only the first was analysed.

The characteristics of patients are shown Table 2. During the study period, we counted 203 (45.2%) patients for whom a request led to a non-admission at least once, and 246 (54.8%) patients immediately admitted (Fig 1). The median age of the cohort was 61 (53–69) years, and 284 patients (63.2%) were men. Totally, 205 patients (45.7%) were hospitalised in the haematology unit, including 56 who came from the bone marrow transplantation (BMT) unit. One hundred seventy (37.8%) patients came from the medical oncology unit, 43 (9.6%) came from in the surgical oncology unit and 31 (6.9%) were directly admitted to ICU from outside the Institute. Among the 203 patients initially not admitted, 54 (26.6%) were admitted at a subsequent request with a median delay from first request of 26 (7–72) h, and 149 (73.4%) were never admitted. Three patients included in the ‘later admission’ group were managed in the ward pending their transfer to another ICU outside the institute. One hundred fifty-nine (78.3%) patients were considered too well to benefit, 36 (17.7%) were considered too sick to benefit and eight (4%) were not admitted because of bed unavailability. The most common reason for requests was a clinical respiratory event present in 92 (45%) patients; in this situation, hypoxemia was present in 66 (72%) patients (Table 1).

**Table 2:** Factors associated with a later ICU admission among initially not admitted patients.

Univariate analysis	« No admission » (n = 149)	« Later Admission » (n = 54)	P value
Age	61 [52–70]	58,5 [51,75–67,5]	0,28
Charlson Comorbidity Index	4 [2–6]	3 [2–6]	0,3
Former surgical procedure ^1^	12 (8,1)	5 (9,3)	0,78
Therapeutic limitation at the first request	11 (7,4)	2 (3,7)	0,52
Former ICU stay ^1^	15 (10,1)	11 (20,4)	0,052
Hematological malignancy VS solid tumor	68 (45,6)	35 (64,8)	0,02
*Acute Leukemia*	27 (18,1)	13 (24,1)	0,35
*Chronic Lymphocytic Leukemia*	5 (3,4)	2 (3,7)	1
*Lymphoma*	23 (15,4)	15 (27,8)	0,046
*Myeloma*	11 (7,4)	1 (1,9)	0,19
*Myelodysplastic syndrome*	2 (1,3)	4 (7,4)	0,049
Level of disease control:			
*Initial phase of treatment*	20 (13,4)	11 (20,4)	0,22
*Ongoing treatment*	6 (4,0)	6 (11,1)	0,06
*Controlled disease*	23 (15,4)	8 (14,8)	0,91
*Complete remission*	13 (8,7)	4 (7,4)	1
*Disease in progression*	83 (55,7)	21 (38,9)	0,03
Allogeneic HSCT	22 (14,8)	10 (18,5)	0,52
Neutropenia	27 (18,1)	16 (29,6)	0,08
Request during off hours	80 (53,7)	27 (50,0)	0,64
Reasons for requests :			
*Clinical respiratory event*	59 (39,6)	33 (61,1)	<0,01
*Hemodynamic event*	64 (43,0)	19 (35,2)	0,32
*Neurological event*	23 (15,4)	8 (14,8)	0,91
*Alteration of renal function or metabolic abnormality*	15 (10,1)	8 (14,8)	0,35
*Warning about a patient likely to deteriorate in the hours or days ahead*	20 (13,4)	2 (3,7)	0,07
*Hypoxemia*	38 (25,5)	28 (51,9)	<0,01
*Shock*	24 (16,1)	11 (20,4)	0,48
*Severe sepsis*	19 (12,8)	7 (13,0)	0,97
*Acute kidney injury*	10 (6,7)	7 (13,0)	0,15
**Multivariate Analysis**	**OR**	**95% CI**	**P value**
Clinical respiratory event	2,6	1,35–5,02	<0,01
Neutropenia	2,25	1,06–4,80	0,03
Former ICU stay^1^	2,75	1,12–6,75	0,03

^1^ A former ICU admission is defined as a recent (< 3 months) ICU admission not during the ingoing hospitalization, HSCT: Hematopoietic stem cell transplantation

Variables are reported as number (percentage) or median [IQR].

### Predictive factors for later ICU admission

Factors associated with later ICU admission among patients initially not admitted are presented in Table 2. Using univariate analysis, former ICU stay, haematologic malignancy, disease control level, neutropenia, a request based on a clinical respiratory event, hypoxemia and a warning about a patient being likely to deteriorate in the hours or days ahead were associated with later ICU admission. By multivariate analysis, a request based on a clinical respiratory event, a former ICU stay and neutropenia were independently associated with later ICU admission.

### Impact of later ICU ADMISSION on outcome

**Fig 2:**
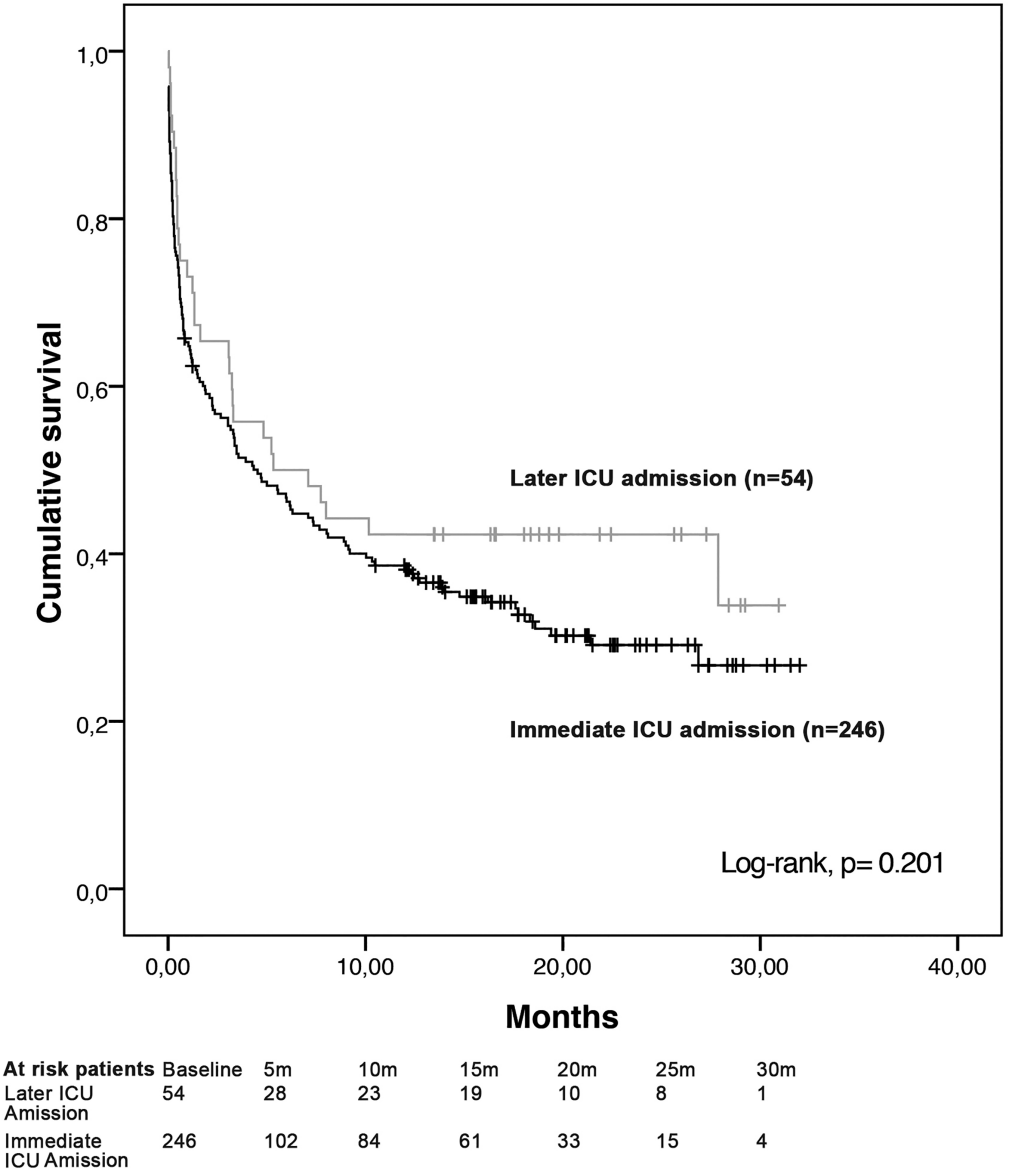
Kaplan-Meier survival curves of « immediate admission » and « later admission » groups.

Demographic data and patient characteristics at ICU admission and during ICU stay were did not differ between the ‘later admission’ and ‘immediate admission’ groups (Table 3). ICU and hospital survival as well as overall survival were similar for the two groups of patients (Fig 2). The median delay from first request to ICU admission was similar among hospital survivors and non-survivors [25 (7–102) h and 28 (10–72) h, respectively; *p* = 0.77). The median follow-up duration was 20 months [95% CI (18–21)]. A later admission was associated with a worsening clinical status of the event that leaded to the first ICU referral in 53 of 54 patients (98%) of the “later admission group”. In this situation the SAPS II score significantly increased between the two referral times (45 (32.25–50.75) vs 47.5 (36.25–57.25), p<0.0001 using Wilcoxon signed rank test).

**Table 3:** Characteristics of ICU stay for « immediate admission » and « late admission » groups.

	« Immediate Admission » (n = 246)	« Later Admission » (n = 54)	P value
Age	62 [54–69]	58,5 [51,75–67,5]	0,23
Male	148 (60,2)	37 (68,5)	0,25
SAPS II *	46 [36–55,75]	47,5 [36,25–57,25]	0,53
Hematology malignancy	151 (61)	34 (64.8)	0.83
Neutropenia	84 (34)	16 (29.6)	0.52
Allogeneic HSCT	54 (22)	10 (18.5)	0.58
Main reasons for ICU admission			
Respiratory failure	135 (54.8)	33 (61.1)	0.19
Shock	54 (21.9)	11 (20.4)	0.79
Severe sepsis	47 (19.1)	7 (13)	0.29
Acute kidney injury	22 (9)	7 (13)	0.36
Support during ICU stay			
Invasive mechanical ventilation*	74 (30,1)	18 (35,3)	0,46
Duration of invasive mechanical ventilation*	4 [1,75–11,25]	4 [1–10]	0,78
Non Invasive Ventilation*	74 (30,1)	20 (39,2)	0,41
High Flow Oxygen Therapy*	45 (18,3)	15 (29,4)	0,07
Norepinephrine*	94 (38,2)	16 (31,4)	0,36
Epinephrine*	11 (4,5)	2 (3,9)	0,61
Renal replacement therapy*	45 (18,3)	13 (25,5)	0,24
Transfusion of blood products*	125 (50,8)	31 (60,8)	0,19
ICU length of stay*	5 [3–10]	6 [4–12]	0,051
ICU length of stay (ICU survivors only)*	5 [3–9]	5,5 [4–12]	0,09
ICU mortality	53 (21.5)	16 (29.6)	0.20
Hospital mortality	64 (26)	18/53 (34)**	0.24

Variables are reported as number (percentage) or median [IQR].

* For ICU parameters, only patients who stayed at the ICU of the institute (n = 51) were described (3 patients transferred in other ICUs were excluded from the analysis, since information regarding ICU treatments was not available).

**Total effective of 53 patients (vital status not known for 1 of the patients transferred to another hospital), HSCT: Hematopoietic stem cell transplantation.

### Factors associated with mortality

**Fig 3:**
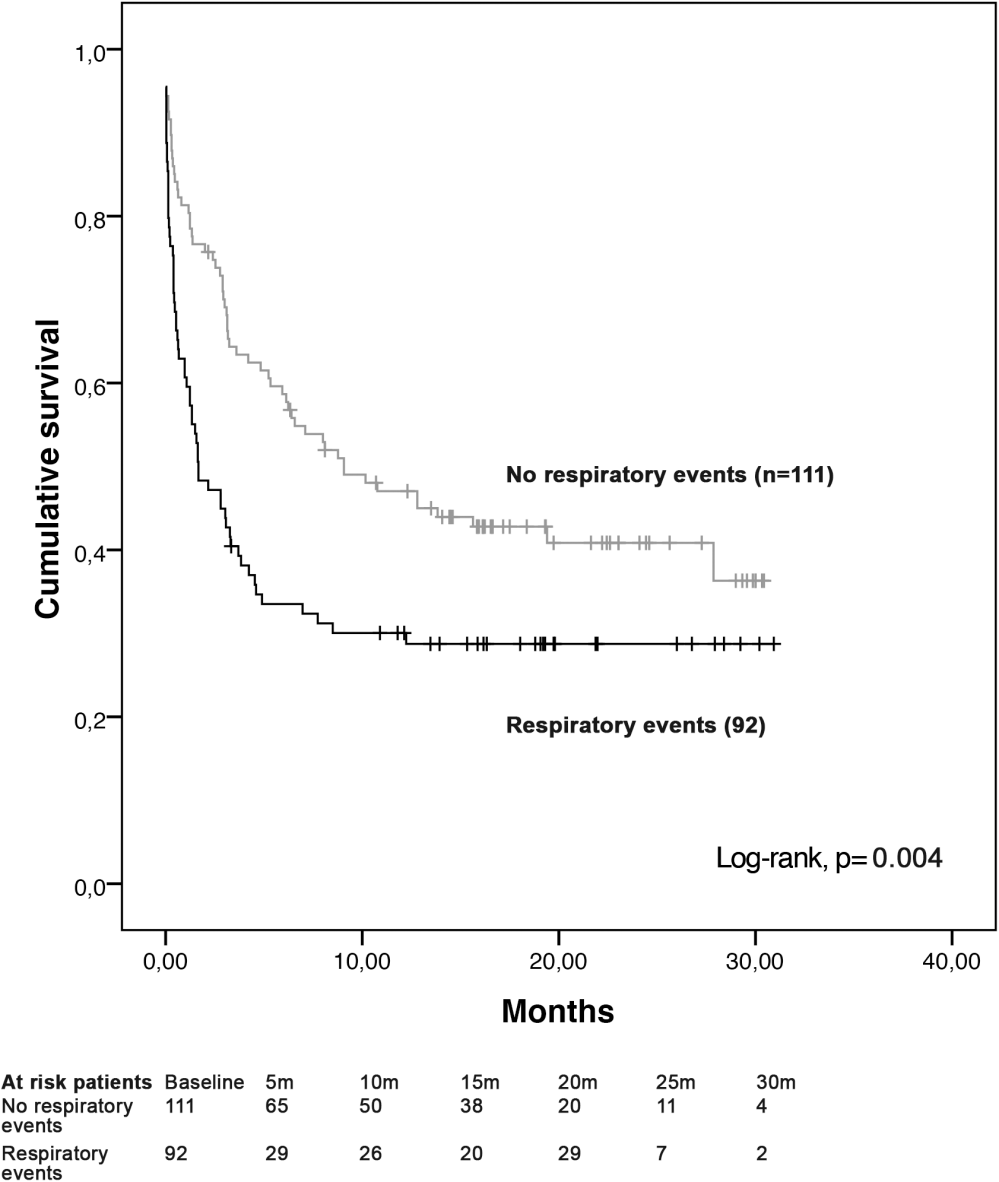
Kaplan-Meier survival curves of patients presenting a respiratory event and no respiratory event.

For patients initially not admitted, hospital mortality was 29.7% (60/202). Factors associated with hospital mortality among patients initially not admitted are shown in Table 4. By multivariate analysis, disease in progression, allogeneic hematopoietic stem cell transplantation (HSCT) and a request based on clinically respiratory events were independently associated with hospital mortality, whereas a request based on severe sepsis was independently associated with survival. Fig 3 displays data on the significantly worse survival of patients for whom the request was based on a respiratory event.

**Table 4:** Characteristics of initially not admitted patients according to hospital mortality.

Univariate analysis	Survivors at hospital discharge (n = 142)	Non-survivors at hospital discharge (n = 60)	P value
Age	59,5 [52–69]	61,5 [53–73,75]	0,53
Charlson Comorbidity Index	3 [2–6]	6 [3–6]	0,03
Former surgical procedure ^1^	16 (11,3)	1 (1,7)	0,03
Former ICU stay ^1^	20 (14,1)	6 (10,0)	0,43
Later ICU admission	35 (25)	18 (30)	0,48
Hematological malignancy n(%) vs solid tumor	74 (52,1)	28 (46,7)	0,48
*Acute Leukemia*	30 (21,1)	10 (16,7)	0,48
*Chronic Lymphocytic Leukemia*	5 (3,5)	2 (3,3)	1
*Lymphoma*	27 (19,0)	13 (21,7)	0,09
*Myeloma*	11 (7,7)	1 (1,7)	0,43
*Myelodysplastic syndrome*	1 (0,8)	2 (3,3)	
Level of disease control :			
*Initial phase of treatment*	24 (16,9)	7 (11,7)	0,35
*Ongoing treatment*	10 (7,5)	2 (3,3)	0,31
*Controlled disease*	25 (17,6)	6 (10,0)	0,17
*Complete remission*	13 (9,2)	3 (5,0)	0,32
*Disease in progression*	62 (43,7)	42 (70,0)	<0,01
Allogeneic HSCT	17 (12,0)	14 (23,3)	0,04
Severe neutropenia	31 (21,8)	12 (20,0)	0,77
Request during off hours	81 (64,6)	25 (41,7)	0,06
Reasons for requests :			
*Clinical respiratory event*	55 (38,7)	37 (61,7)	<0,01
*Hemodynamic event*	60 (42,3)	22 (36,7)	0,46
*Neurological event*	18 (12,7)	13 (21,7)	0,1
*Alteration of renal function or metabolic abnormality*	17 (12,0)	6 (10,0)	0,69
*Warning about a patient likely to deteriorate in the hours or days ahead*	17 (12,0)	5 (8,3)	0,45
*Hypoxemia*	39 (27,5)	27 (45,0)	0,02
*Hypotension*	26 (18,3)	9 (15,0)	0,57
*Severe sepsis*	23 (16,2)	3 (5,0)	0,04
*Acute kidney injury*	11 (7,7)	6 (10,0)	0,6
Multivariate analysis	OR	95% CI	P value
Disease in progression	3,15	1,6–6,19	<0,01
Allogeneic HSCT	2,5	1,06–5,89	0,04
Clinical respiratory event	2,36	1,22–4,56	0,01
Severe sepsis	0,27	0,08–0,99	0,049

Variables are reported as number (percentage) or median [IQR], HSCT: Hematopoietic stem cell transplantation

Total effective of 202 patients (vital status not known for 1 of the patients transferred to another hospital)

^1^ A former ICU admission is defined as a recent (< 3 months) ICU admission not during the ingoing hospitalization

### Outcome of patients never admitted

Of the 149 patients who were never admitted between the first denied ICU admission and hospital discharge, 31 (20.8%) were judged too sick to benefit, 114 (76.5%) were judged too well to benefit and four (2.6%) were not admitted because of bed unavailability. The survival rate of the patients never admitted because they were judged too sick to benefit was 25.8% (8/31 patients) at hospital discharge, while it was 12.9% (4/31 patients) after 90 days.

The mean survival of patients judged too well to benefit from ICU admission was significantly longer than that of patients judged too sick to benefit and was 443 days (95% CI, 368–517) and 38 days (95% CI, 6–71), respectively (*p* < 0.0001, using log-rank test).

## Discussion

We reported a 20-month study of 203 hospitalised cancer patients who were referred and denied admission at least once to ICU. Twenty-seven percent of these patients initially not admitted were admitted at a later time. To the best of our knowledge, this study is the first designed to determine factors that are associated with hospital mortality and that present at the time of the first referral among patients initially not admitted to ICU and factors predictive of later ICU admission in such a situation. Factors independently associated with an increased risk of a later ICU admission among patients initially not admitted included a former ICU stay, a request based on a clinical respiratory event and neutropenia. For this population, the overall hospital mortality rate was 30% and associated with malignant disease in progression, a request based on a clinical respiratory event and a history of allogeneic HSCT. Compared with immediate ICU admission, later ICU admission was not associated with hospital mortality.

In accordance with previous studies [3,9,10], we showed a non-admission rate of 45%, while the main cause of non-admission to ICU was a too well to benefit consideration. The most common reason for requests was a clinical respiratory event (present in 45% of the patients). A request based on a clinical respiratory event was an independent risk factor for both a later ICU admission and hospital mortality. It has been shown that in ICU cancer patients, hypoxemia [11], acute respiratory failure [4,12] and the need for invasive mechanical ventilation [13–15] are associated with a poor outcome. Along these lines, isolated respiratory symptoms such as polypnoea in leukemic patients hospitalised in wards appear to be predictive of serious clinical events [16] and outcome when appearing during the chemotherapy induction period [17]. In the present study, patients presenting with clinical respiratory events were hypoxemic in approximately 70% of the cases. This underlines the need for improving the management strategy of clinical respiratory events in cancer patients hospitalised in wards. A more precise evaluation of respiratory events could be performed, including radiological criteria with a view to more accurately assessing risk factors for the pejorative evolution of respiratory symptoms. It has been shown that the radiological extension of lung infiltrates is associated with a poor outcome in the context of ARF[18,19]. We recently described [20] a significant increase in mortality rates in cancer patients admitted to ICU for more than two days after the onset of respiratory symptoms and a trend towards a worse outcome when at least three pathologic quadrants were observed on chest X-ray at ICU admission. Further studies are warranted to assess the potential benefit of an early admission on moderate clinical respiratory events including clinical and radiological evaluation criteria. Importantly, we showed that neutropenic patients represented a high-risk population for subsequent ICU admission after the first denial. Neutropenic patients represent up to 30% of critically ill cancer patients admitted to ICU [4], and recent studies strongly suggest that ICU admission denial based on neutropenia should be discouraged as they failed to demonstrate the impact of neutropenia on outcome [21]. In such conditions, accurate clinical evaluation is difficult because clinical inflammatory symptoms appear to be attenuated due to the lack of neutrophils and might explain failure to appropriately evaluate a patient’s clinical status. To this end, neutropenia recovery represents a high-risk period during which the patient clinical status is likely to be worsened [22]. Neutropenia recovery silently occurs in the vast majority of patients; [22] however, deterioration in respiratory status has been reported during the resolution of leukopenia[23,24]. In haematology patients, the suboptimal evaluation of wards may result in the undervaluation of disease severity followed by a clinical deterioration [25].

Compared with an immediate ICU admission, a subsequent ICU admission occurring after at least one denial was not associated with prognosis. On the contrary, previous studies have described an association between increased mortality rates and time from physiological derangement to ICU admission [1,4,26] or delayed ICU admission after the first refusal [3,10,27,28]. In these situations, the main cause of refusal was the lack of bed availability [10,27,28]. In contrast, most of our patients denied ICU admission were considered too well to benefit. Similarly, O’Callaghan et al. [29] and Garrouste-Orgeas et al. [9,30] did not observe any impact on mortality between patients admitted immediately and later. In these situations, refusal for ICU admission is related to the number of beds available and also to the ability of the triaging physician to examine the patient and ICU physician seniority [9]. These findings suggest that the current practice of the Institute’s RRT triage strategy is appropriate because decisions to admit are always made by the senior intensivist in close collaboration with the referring haematologist/oncologist and after the systematic clinical examination of each proposed patient.

In contrast to previous studies that showed increased mortality in patients admitted to ICU with severe sepsis [11,31], a request based on severe sepsis was associated with a lower hospital mortality rate in our study. Nevertheless, we studied prognostic factors existing prior to an eventual ICU admission. Our study population comprising patients initially denied admission to ICU mainly because of a too well to benefit assessment, makes our results difficult to compare with those obtained from the analysis of ICU cancer patients. However, we did not identify any negative impact on the outcome of patients referred for severe sepsis and initially denied admission, and this suggests a good triage policy and adequate management of patients with haemodynamic instability in the ward. An awareness campaign has been led by intensivists of the institute based on the recognition of the warning sign ‘hypotension’ and informing physicians about the need for the early treatment of severe sepsis in the ward in close collaboration with intensivists and how to do so. Moreover, the patients who met ICU admission criteria were immediately admitted and were therefore not included in the analysis. Among haemodynamic requests, the low rate of cardiac arrests should suggest the beneficial impact of the RRT system [32,33], although we did not perform any before and after study in our centre. Surprisingly, in the present study neurologic events were not associated with prognosis. Accordingly, we have recently shown in a prospective multicentre observational study including 1011 haematology patients admitted to the ICU that neurologic events are not associated with outcomes [4]. In these situations, neurologic dysfunctions are commonly associated with metabolic disorders or seizures which are known to be associated with a favourable evolution.

As a previous study has reported an increased risk of mortality for patients admitted during off hours [34], we analysed if the time of request impacted patient outcome. We did not observe any difference in triage and hospital mortality among patients referred during off hours. Bone marrow transplantation is already known to be a major prognostic factor in critically ill cancer patients [4,11,12]. Consistent with previous studies, we reported a history of allogeneic bone marrow transplantation as an independent risk factor for hospital mortality, whereas the type of underlying malignancy did not influence the outcome of our study population [12,35,36]. Similar to Thiéry et al [3], the fact that hospital survival in patients never admitted because they were considered too sick to benefit from ICU admission was 25.8% suggests inaccurate clinical judgment by the intensivist. We conducted a further analysis and observed that only 13.8% were alive after 90 days and that survival rates dramatically decreased towards the end of the follow-up. It is uncertain whether there would be any benefit from an ICU stay for these patients.

Some limitations of our study should be considered. First, the study was performed at a single centre, and RRT role and triage may vary across hospitals. However, our admission rates were similar to those reported in other studies, and all RRT members were senior intensivists from the institute with experience in the management of cancer patients. Second, the comparison of the clinical status at the time of the first referral between patients never admitted and those later admitted could have been of critical importance to better investigate the factors associated with later admission. Unfortunately, we were not able to realize this comparison, due to the initial design of the study. Third, there are different numbers of RRT doctors who are on duty between off hours and office hours (1 vs 3, respectively), thus results should be interpreted cautiously since a statistical bias may be present in this situation. However, a request during off hours was not associated with a subsequent ICU admission or prognosis.

## Conclusion

In summary, 45% of the patients for whom RRT was requested were initially not admitted to ICU, mainly because of being evaluated as too well to benefit from ICU admission. Among these, up to a quarter were admitted at a later time. Compared with an immediate ICU admission, a subsequent ICU admission occurring after at least one denied admission was not associated with prognosis. An ICU request for a clinical respiratory event was an independent risk factor for both later ICU admission and hospital mortality. To assess the potential benefit of early ICU admission for moderate clinical respiratory events in hospitalised cancer patients, a prospective study is warranted.

## Supporting information

S1 File (XLS)
